# Differential Expression of LncRNA MIAT and Its Clinical Significance in Intracranial Aneurysms

**DOI:** 10.1002/brb3.70500

**Published:** 2025-05-05

**Authors:** Fuquan Liu, Shuai Hao, Jingliyu Wang, Lei Chen, Ning Jiang, Laixing Liu, Xiangyi Wang

**Affiliations:** ^1^ Department of Neurosurgery, Mental Health Institute of Inner Mongolia Autonomous Region The Third Hospital of Inner Mongolia Autonomous Region Hohhot China; ^2^ Department of Neurosurgery Inner Mongolia Baogang Hospital Baotou China

**Keywords:** intracranial aneurysm, LncRNA MIAT, RIA, ROC, serum, UIA

## Abstract

**Purpose:**

Intracranial aneurysm (IA) is characterized by localized dilation or ballooning of a blood vessel in the brain with life‐threatening consequences. This study set out to investigate the value of Long noncoding RNA myocardial infarction associated transcript (LncRNA MIAT) in diagnosis, rupture prediction, and prognosis for IA patients.

**Methods:**

The clinical characteristics of controls (100 cases) and IA patients, including 88 cases of ruptured IA (RIA) and 132 cases of unruptured IA (UIA), were analyzed using the chi‐square test and *t*‐test. MIAT expression in the serum was detected. The diagnostic efficacy of MIAT for IA was assessed through receiver operating characteristic (ROC) curve analysis. IA patients were categorized into low‐expression and high‐expression groups, and the correlation between MIAT expression and clinical indicators in IA patients was analyzed. The risk factors for IA rupture were analyzed. The correlation between MIAT expression and the overall survival of IA patients was assayed. The risk factors for poor prognosis in IA patients were analyzed.

**Results:**

LncRNA MIAT was elevated in IA patients, with higher expression in RIA patients than in UIA patients. MIAT expression could distinguish between healthy control and IA patients (area under the curve, AUC = 0.794, cut‐off value = 1.26, sensitivity = 68.64%, specificity = 83.00%, *p* < 0.001), healthy control and UIA patients (AUC = 0.782, cut‐off value = 1.15, sensitivity = 75.76%, specificity = 68.00%, *p* < 0.001), and UIA patients and RIA patients (AUC = 0.690, cut‐off value = 1.46, sensitivity = 76.14%, specificity = 68.94%, *p* < 0.001). MIAT expression was significantly associated with hypertension, IA location, and IA rupture status. MIAT was an independent risk factor for IA rupture. For every one‐unit increase in the relative expression of MIAT, the risk of poor prognosis in IA patients increased by 2.415 times (*p* = 0.014, 95% CI: 1.192–4.890). MIAT expression was correlated with the overall survival of IA patients and IA rupture in UIA patients. MIAT was an independent risk factor for poor prognosis of IA patients. For every one‐unit increase in the relative expression of MIAT, the risk of poor prognosis in IA patients increased by 2.415 times (*p* = 0.014, 95% CI: 1.192–4.890).

**Conclusion:**

Highly expressed MIAT is an independent risk factor for poor prognosis of IA and can support IA diagnosis and rupture prediction.

## Graphical abstract

Increased expression of LncRNA MIAT is associated with an elevated risk of intracranial aneurysm rupture.

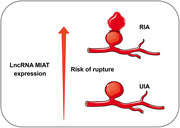

## 1 Introduction

Intracranial aneurysm (IA) refers to the pathological dilation of the cerebral arteries and the wall thinning of intracranial arteries and represents a highly complex challenge in modern neurology and neurosurgery (Freneau et al. 2022). IA carries a high risk of disability and mortality, and if the aneurysm ruptures, it can result in severe outcomes including nontraumatic subarachnoid hemorrhage (Gareev et al. 2020). As most IA are asymptomatic before rupture, early detection of IA is crucial for effective monitoring of their progression and implementation of preventive measures (Brinjikji et al. 2016). Digital subtraction angiography (DSA) is currently the best method for diagnosing IA (Sebok et al. 2021). However, DSA is invasive and cannot visualize occlusion of distal blood vessels, and the high cost and potential risks associated with injecting iodine contrast agents make DSA unsatisfactory for early screening and treatment of IA (Grossberg, Howard, and Saindane 2019). Therefore, treatment and prevention of IA remain a major challenge.

Long noncoding RNAs (LncRNAs) are extensively implicated in a range of cardiovascular diseases, such as atherosclerosis, myocardial infarction, and hypertension (Zhang et al. 2019). These cardiovascular diseases represent significant risk factors for IA development and are related to dysregulated LncRNA expression (Gareev et al. 2020). In addition, the abnormal expression of LncRNA is closely associated with the pathogenesis of IA (H. Li et al. 2017). Of note, LncRNAs can serve as biomarkers and have the potential for diagnosing and predicting IA (Sun et al. 2020). Therefore, identifying LncRNAs that can serve as biomarkers for IA may be a promising therapeutic approach.

Myocardial infarction‐associated transcript (MIAT), recognized as one of the lncRNAs in 2006, is strongly correlated with the occurrence of myocardial infarction and microvascular dysfunction (Liao et al. 2016). For instance, suppression of LncRNA MIAT expression alleviates apoptosis of human aortic endothelial cells and reduces atherosclerosis (T. Li et al. 2021). Knocking down MIAT can inhibit the proliferation and migration of endothelial cells and alleviate microvascular dysfunction in diabetes (Yan et al. 2015). More importantly, MIAT is upregulated in IA and increases endothelial cell apoptosis and vascular injury (X. Li, Zhao, Liu, and Tong 2020). Further exploration of the differential expression of MIAT in IA and its clinical significance plays a crucial role in the diagnosis and prognosis of IA.

In this study, we included IA patients and healthy controls to investigate the role of MIAT expression in the serum of IA patients and its value in the diagnosis, progression, and prognosis of IA, providing potential therapeutic directions for the treatment of IA.

## 2 Materials and Methods

### 2.1 Study Population

A total of 220 patients diagnosed with IA at The Third Hospital of Inner Mongolia Autonomous Region from January 2016 to December 2018 and 100 healthy controls (who underwent health examinations at The Third Hospital of Inner Mongolia Autonomous Region during the same period) were included in our study. All IA patients underwent computed tomography (CT) scans to assess whether the aneurysm had ruptured. Based on the CT scan images, IA patients were assigned to unruptured IA (UIA) group (132 cases) and ruptured IA (RIA) group (88 cases).

### 2.2 Inclusion and Exclusion Criteria

The inclusion criteria for control individuals were as follows: (1) no statistically significant differences in age and gender compared to IA patients; (2) no history of inflammatory‐related diseases, cerebrovascular‐related diseases, tumors, or immune‐related diseases.

The inclusion criteria for IA patients were as follows: (1) age ≥ 18 years; (2) diagnosed with IA by DSA, magnetic resonance angiography, or three‐dimensional CT angiography.

The exclusion criteria were as follows: (1) age < 18 years; (2) incomplete clinical data; (3) mycotic, traumatic, or fusiform aneurysms; (4) history of autoimmune or rheumatic diseases; (5) concurrent systemic infections; (6) pregnancy.

### 2.3 Data Collection and Follow‐Up Investigation

Blood samples were collected from all participants and centrifuged at 1500 g/min for 15 min to remove blood cells. After that, the supernatant was transferred to new centrifuge tubes and further centrifuged at 10,000 g/min for 10 min to remove cellular debris. The obtained serum samples were stored at –80°C. Information such as gender, age, height, weight, alcohol‐consumption, and smoking status of all participants was recorded. Serum levels of total cholesterol (TC), high‐density lipoprotein cholesterol (HDL‐C), and low‐density lipoprotein cholesterol (LDL‐C) were measured using conventional methods. Hypertension was diagnosed when patients had systolic blood pressure ≥ 140 mmHg, diastolic blood pressure ≥ 90 mmHg, or were receiving antihypertensive treatment. Diabetes was diagnosed when the fasting blood glucose level was ≥ 7.0 mmol/L or when the patients were receiving oral hypoglycemic agents or insulin therapy.

IA patients were followed up for 63 months, and the survival of IA patients after treatment and the occurrence of IA rupture in UIA patients were recorded. There were no statistically significant differences in age, gender, and other parameters between the groups.

### 2.4 Quantitative Real‐Time Polymerase Chain Reaction (qRT‐PCR)

Total RNA was extracted using TRIzol reagent (15596018CN, Invitrogen, Carlsbad, CA, USA). The RNA was reverse transcribed into cDNA using the PrimeScript RT reagent Kit (RR037B, Takara, Dalian, China). Subsequently, qRT‐PCR was performed using TB Green Premix Ex Taq II (RR820B, Takara) on the CFX96 Real‐Time PCR Detection System (Biorad, Hercules, CA, USA). Data were analyzed using the 2^−ΔΔCt^ method (Livak and Schmittgen 2001), with β‐actin as an internal reference. The primers were synthesized by Sangon Biotech (Shanghai, China). The primer sequences are listed in Table 1.

**TABLE 1:** qRT‐PCR primer sequences.

Gene	Forward 5’‐3’	Reverse 5’‐3’
MIAT	TATTTGCAGGGGGTGCTCTG	GGGCAGGGGGTCTAACTCTA
β‐actin	ACGACATGGAGAAGATCTG	TGTTGAACGTCTCGAACATG

### 2.5 Statistical Analysis

SPSS22.0 software (IBM SPSS Statistics, Armonk, NY, USA) and GraphPad Prism 8.0 software (GraphPad Software Inc., San Diego, CA, USA) were used for data analyses. The data were categorized into categorical and continuous variables. Categorical data were expressed as frequencies and percentages, while continuous variables were presented as mean ± standard deviation. Normality was assessed using the Kolmogorov–Smirnov and D'Agostino tests. For normally distributed continuous variables, independent sample *t*‐tests were used for comparisons between groups, and one‐way analysis of variance (ANOVA) was used for comparisons among multiple groups, followed by Tukey's multiple comparisons test. Comparisons of categorical data between groups were conducted using the chi‐square test. The diagnostic efficacy of parameters was evaluated using receiver operator characteristic (ROC) curve analysis to obtain cutoff values. The risk factors for IA rupture were assessed using logistic regression analysis. Initially, univariate analysis was conducted for each risk factor, and factors with *p* < 0.1 were included in the multivariate logistic regression analysis. The influence of MIAT expression on IA prognosis was analyzed using the log‐rank test and Kaplan–Meier curve analysis. Additionally, the impact factors for IA prognosis were evaluated using COX regression analysis. The univariate analysis was performed for each risk factor, and factors with *p* < 0.1 were included in the multivariate COX regression analysis. All tests were two‐tailed, with *p* < 0.05 considered statistically significant.

## 3 Results

### 3.1 Clinical Characteristics of the Study Population

In this study, there were no statistically significant differences in age, gender, body mass index (BMI), hypertension, TC, HDL‐C, LDL‐C, diabetes, smoking, and alcohol consumption between IA patients (*n* = 220) and control individuals (*n* = 100) (Table 2, all *p* > 0.05).

**TABLE 2:** Comparison of clinical characteristics.

	Control (*N* = 100)	IA (*N* = 220)	*p*	
**Age (years)**	53.16 ± 7.67	53.43 ± 7.31	0.765^a^	
**Gender (female)**	61 (61.0%)	133 (60.5%)	0.926^b^	
**BMI (kg/m^2^)**	24.11 ± 1.70	23.93 ± 1.79	0.419^a^	
**Hypertension**	47 (47.0%)	105 (47.7%)	0.904^b^	
**TC (mmol/L)**	4.36 ± 0.92	4.54 ± 0.85	0.091^a^	
**HDL‐c (mmol/L)**	1.35 ± 0.45	1.37 ± 0.27	0.713^a^	
**LDL‐c (mmol/L)**	2.50 ± 0.71	2.44 ± 0.63	0.484^a^	
**Diabetes**	16 (16.0%)	47 (21.4%)	0.263^b^	
**Alcohol, current**	31 (31.0%)	73 (33.2%)	0.699^b^	
**Smoking, current**	21 (21.0%)	61 (27.7%)	0.201^b^	
**Size of aneurysm (mm)**	—	8.84 ± 2.06	—	
**Location of aneurysm**				
ICA	—	61 (27.7%)	—	
MCA	—	78 (35.5%)	—	
ACA/Pcom/posterior	—	81 (36.8%)	—	
**Aneurysm ruptured**	—	88 (40.0%)	—

BMI: body mass index; TC: total cholesterol; HDL‐c: high‐density lipoprotein‐cholesterol; LDL‐c: low‐density lipoprotein‐cholesterol; ICA: internal carotid artery; MCA: middle cerebral artery; ACA: anterior cerebral artery; Pcom: posterior communicating artery; posterior: posterior circulation (including the vertebral artery, basilar artery, cerebellar arteries, and posterior cerebral artery).

*Note*: The data are presented as mean ± standard deviation or *n* (%). *p* < 0.05 was considered statistically significant.

^a^Analyzed by independent sample *t*‐tests.

^b^Analyzed by chi‐square test.

### 3.2 MIAT Is Overexpressed in the Serum of IA Patients and Has High Diagnostic Value for IA

**FIGURE 1:**
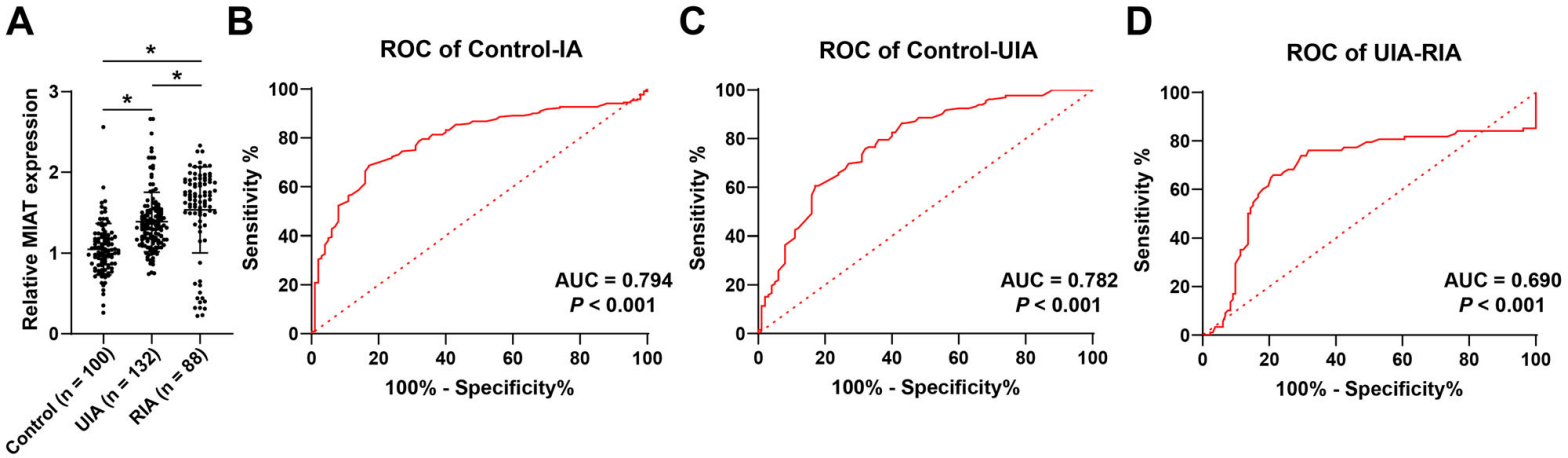
**MIAT is overexpressed in the serum of IA patients and has high diagnostic value for IA**. (A) The expression of MIAT in the serum of all participants was detected by qRT‐PCR. (B) The diagnostic efficacy of MIAT in distinguishing IA from controls was analyzed by ROC curve. (C) The diagnostic efficacy of MIAT in distinguishing UIA from controls was analyzed by ROC curve. (D) The diagnostic efficacy of MIAT in distinguishing between UIA and RIA was analyzed by ROC curve. Data in panel A were analyzed by one‐way ANOVA, followed by Tukey's multiple comparisons test. Data in panels B–D were analyzed by ROC curve, *p < 0.05.

A total of 220 IA patients and 100 healthy controls were included. IA patients were classified as UIA (*n* = 132) and RIA (*n* = 88). The qRT‐PCR analysis showed that compared to the control group, MIAT expression in the serum of both UIA and RIA patients was significantly elevated, with RIA patients showing remarkably higher MIAT expression than UIA patients (*p* < 0.05, Figure 1A). ROC curves were plotted to further investigate the diagnostic value of MIAT expression for IA. In discriminating IA patients from control individuals, the area under the curve (AUC) of MIAT was 0.794, with a cutoff value of 1.26, sensitivity of 68.64%, and specificity of 83.00% (*p* < 0.05, Figure 1B). In discriminating UIA patients from control individuals, the AUC of MIAT was 0.782, with a cutoff value of 1.15, sensitivity of 75.76%, and specificity of 68.00% (*p* < 0.05, Figure 1C). In discriminating UIA from RIA patients, the AUC of MIAT was 0.690, with a cutoff value of 1.46, sensitivity of 76.14%, and specificity of 68.94% (*p* < 0.05, Figure 1D). These results indicate that MIAT expression levels have certain diagnostic value for discriminating between UIA and RIA.

### 3.3 Correlation Analysis of MIAT Expression With Clinical Pathological Features of IA Patients

We then analyzed the relationship between MIAT expression and clinical pathological features of IA patients. IA patients were categorized into high‐ and low‐expression groups based on the median MIAT expression in the serum of IA patients. The results showed that MIAT expression was significantly associated with hypertension, aneurysm location, and IA rupture status (*p* < 0.05), with no significant associations of MIAT with age, gender, BMI, TC, HDL‐C, LDL‐C, diabetes, smoking, alcohol consumption, and aneurysm size (*p* > 0.05) (Table 3).

**TABLE 3:** The relationship between serum MIAT expression and clinical pathological features of IA patients.

Clinical pathological features	MIAT		*p*
	Low expression (*N* = 110)	High expression (*N* = 110)	
**Age (years)**	53.56 ± 6.94	53.29 ± 7.70	0.783^a^
**Gender (female)**	67 (60.9%)	66 (60.0%)	0.890^b^
**BMI (kg/m^2^)**	23.93 ± 1.77	23.94 ± 1.81	0.967^a^
**Hypertension**	43 (39.1%)	62 (56.4%)	0.010^b^
**TC (mmol/L)**	4.57 ± 0.85	4.52 ± 0.85	0.674^a^
**HDL‐c (mmol/L)**	1.35 ± 0.29	1.39 ± 0.24	0.361^a^
**LDL‐c (mmol/L)**	2.46 ± 0.61	2.43 ± 0.66	0.778^a^
**Diabetes**	24 (21.8%)	23 (20.9%)	0.869^b^
**Alcohol, current**	40 (36.4%)	33 (30.0%)	0.316^b^
**Smoking, current**	31 (28.2%)	30 (27.3%)	0.880^b^
**Size of aneurysm (mm)**	8.77 ± 2.17	8.91 ± 1.95	0.602^a^
**Location of aneurysm**			0.006^b^
ICA	40 (36.4%)	21 (19.1%)	
MCA	39 (35.5%)	39 (35.5%)	
ACA/Pcom/posterior	31 (28.2%)	50 (45.5%)	
**Aneurysm ruptured**	21 (19.1%)	67 (60.9%)	< 0.001^b^

*Note*: The data are presented as mean ± standard deviation or *n* (%). *p* < 0.05 was considered statistically significant.

^a^Analyzed by independent sample *t*‐tests.

^b^Analyzed by chi‐square test.

### 3.4 MIAT Is Independently Associated With the Occurrence of IA Rupture

To further investigate whether MIAT is independently associated with IA rupture, we performed logistic regression analysis with IA rupture at enrollment as the dependent variable and various clinical indicators as independent variables. Firstly, univariable logistic regression analysis was performed, and then hypertension (*p* < 0.1), aneurysm location (*p* < 0.1), aneurysm size (*p* < 0.1), and MIAT expression (*p* < 0.1) were included as independent variables in the multivariate logistic regression analysis. The results showed that aneurysm location (MCA vs. ICA: *p* = 0.940, OR: 1.031, 95% CI: 0.463–2.297;ACA/Pcom/posterior vs. ICA: *p* = 0.017, OR: 2.589, 95% CI: 1.181–5.674), aneurysm size (*p <* 0.001, OR: 1.417, 95% CI: 1.203–1.669), and MIAT expression were independent risk factors for IA rupture (*p* < 0.05, Table 4), and for each unit increase in relative MIAT expression, the risk of IA rupture increased by 2.145 times (*p* = 0.029, OR: 2.145, 95% CI: 1.082–4.254).

**TABLE 4:** MIAT is independently associated with the occurrence of IA rupture.

	Univariable logistic			Multivariable logistic		
	*p*	OR	95% CI	*p*	OR	95% CI
**Age** (years)	0.684	0.992	0.956–1.030	—	—	—
**Gender** (female)	0.736	1.100	0.634–1.907	—	—	—
**BMI** (kg/m2)	0.134	0.889	0.762–1.037	—	—	—
**Hypertension**	0.055	1.705	0.990–2.938	0.219	1.461	0.798–2.673
**TC** (mmol/L)	0.102	0.764	0.552–1.055	—	—	—
**HDL‐c** (mmol/L)	0.985	0.991	0.362–2.714	—	—	—
**LDL‐c** (mmol/L)	0.341	0.811	0.526–1.248	—	—	—
**Diabetes**	0.946	1.023	0.530–1.973	—	—	—
**Alcohol, current**	0.815	1.071	0.604–1.896	—	—	—
**Smoking, current**	0.854	1.058	0.580–1.930	—	—	—
**Size of aneurysm** (mm)	< 0.001	1.431	1.227–1.669	< 0.001	1.417	1.203–1.669
**Location of aneurysm**	< 0.001	—	—	0.012	—	—
MCA vs. ICA	0.203	1.624	0.770–3.425	0.940	1.031	0.463–2.297
ACA/Pcom/posterior vs. ICA	< 0.001	4.030	1.942–8.363	0.017	2.589	1.181–5.674
**MIAT expression**	0.017	2.192	1.152–4.168	0.029	2.145	1.082–4.254

*Note*: *p* < 0.05 was considered statistically significant.

OR: odds ratio; CI: confidence interval.

### 3.5 Highly Expressed MIAT Is Associated With Poor Prognosis of IA Patients

**FIGURE 2:**
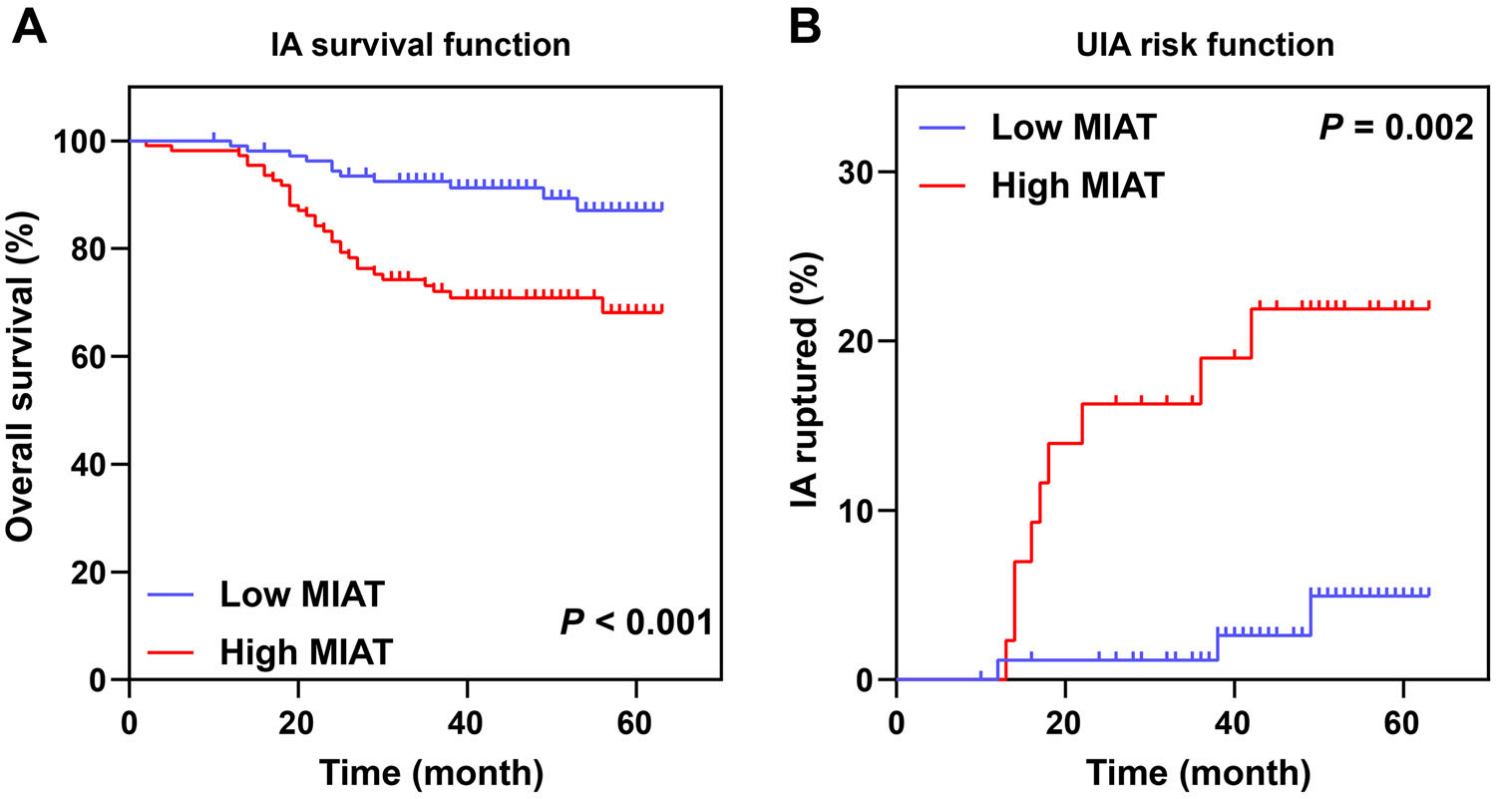
**Highly expressed MIAT is associated with poor prognosis of IA patients**. (A) The impact of MIAT expression on the overall survival of IA patients was assessed by Kaplan–Meier analysis. (B) The influence of MIAT expression on the occurrence of IA rupture in UIA patients was evaluated by Kaplan–Meier analysis.

The patients were categorized into high‐ and low‐expression groups based on the median MIAT expression in the serum of IA patients, followed by comparison of overall survival. Kaplan–Meier analysis showed that IA patients with high MIAT expression had a decreased overall survival compared to those with low MIAT expression (*p* < 0.05, Figure 2A). Additionally, the incidence of IA rupture was increased in UIA patients with high MIAT expression compared to those with low MIAT expression (*p* < 0.05, Figure 2B).

To further investigate whether MIAT was independently associated with poor prognosis in IA patients, we used the occurrence of poor prognosis as the dependent variable, defined as death or the occurrence of IA rupture during the follow‐up investigation. The log‐rank test was performed to select variables with *p* < 0.1, including smoking, aneurysm location, aneurysm size, and MIAT expression, as independent variables for the multivariate COX regression analysis. The results showed that aneurysm location (MCA vs. ICA: *p* = 0.559, HR: 0.730, 95% CI: 0.254–2.096; ACA/Pcom/posterior vs. ICA: *p* = 0.041, HR: 2.600, 95% CI: 1.038–6.510), aneurysm size (*p* < 0.001, HR: 1.344, 95% CI: 1.170–1.544), and MIAT expression were independent risk factors for poor prognosis in IA patients (*p* < 0.05, Table 5), and for each unit increase in relative MIAT expression, the risk of poor prognosis of IA patients increased by 2.415 times (*p* = 0.014, HR: 2.415, 95% CI: 1.192–4.890).

**TABLE 5:** MIAT expression is independently associated with poor prognosis in IA patients.

	Univariable COX			Multivariable COX		
	*p*	HR	95% CI	*p*	HR	95% CI
**Age** (years)	0.191	1.028	0.986–1.071	—	—	—
**Gender** (female)	0.201	0.670	0.363–1.238	—	—	—
**BMI** (kg/m2)	0.550	1.051	0.893–1.236	—	—	—
**Hypertension**	0.313	1.345	0.757–2.390	—	—	—
**TC** (mmol/L)	0.190	0.801	0.575–1.116	—	—	—
**HDL‐c** (mmol/L)	0.675	0.794	0.269–2.337	—	—	—
**LDL‐c** (mmol/L)	0.804	0.944	0.598–1.490	—	—	—
**Diabetes**	0.673	1.157	0.589–2.273	—	—	—
**Alcohol, current**	0.404	0.762	0.402–1.444	—	—	—
**Smoking, current**	0.078	1.699	0.943–3.059	0.147	1.551	0.857–2.808
**Size of aneurysm** (mm)	< 0.001	1.414	1.238–1.615	< 0.001	1.344	1.170–1.544
**Location of aneurysm**	< 0.001	—	—	0.002	—	—
MCA vs. ICA	0.742	1.190	0.423–3.343	0.559	0.730	0.254–2.096	
ACA/Pcom/posterior vs. ICA	0.001	4.674	1.953–11.186	0.041	2.600	1.038–6.510	
**MIAT expression**	0.005	2.494	1.308–4.754	0.014	2.415	1.192–4.890	

*Note*: *p* < 0.05 was considered statistically significant.

HR: hazard ratio.

## 4 Discussion

As a vascular disease, UIA may be asymptomatic for many years, but once ruptured, it can lead to subarachnoid hemorrhage with a high mortality rate and severe consequences, including immediate death in 12% of patients, over 30% mortality within one month, and 25% to 50% mortality within six months (Pontes et al. 2021). However, there is a lack of effective tools and indicators to detect IA rupture. Hence, discovering biomarkers for the diagnosis of IA is critical for early prevention and intervention. Herein, we included 88 patients with RIA, 132 patients with UIA, and 100 healthy controls to analyze the impact of MIAT expression on the rupture and adverse outcomes of IA.

In the present study, we found that compared with healthy controls, MIAT was highly expressed in patients with IA. Consistently, recent studies have found that MIAT contributes to aortic diseases. For example, MIAT is significantly elevated in bicuspid aortic valve aortopathy and is associated with dilated aortas (Lim et al. 2022). In thoracic aortic aneurysm, MIAT is overexpressed and promotes the activity of aortic vascular smooth muscle cells (Chen et al. 2019). LncRNA MIAT is upregulated in atherosclerosis and induces apoptosis in human aortic endothelial cells (T. Li et al. 2021). Damage to endothelial cells leads to impaired vascular repair and angiogenesis, which can be alleviated by inhibiting MIAT expression (Li et al. 2021; Ramirez‐Velandia et al. 2024). Notably, silencing LncRNA MIAT expression can alleviate vascular deformation, reduce endothelial apoptosis, and prevent the occurrence of IA (X. Li et al. 2020). In addition, MIAT can serve as a potential biomarker to distinguish ischemic stroke patients from healthy controls, with ischemic stroke patients showing poor prognosis when MIAT is highly expressed (Zhu et al. 2018). MIAT is significantly elevated in the serum of patients with coronary heart disease and holds potential for the diagnosis and prognosis prediction of coronary heart disease (Tan et al. 2019). On the above‐mentioned observation, we assumed that MIAT may be involved in the development of IA and have the potential to serve as a biomarker in the diagnosis of IA. Here, the ROC curve analysis showed that MIAT had diagnostic power for IA, and MIAT may differentiate between UIA and RIA patients. The specificity (68%) and sensitivity (76.14%) of MIAT in differentiating between UIA and RIA still require further improvement, which might be achievable by integrating other biomarkers for comprehensive diagnosis in our future research. These findings suggested that MIAT has a high diagnostic value for IA and may be considered a useful predictor in differentiating between RIA and UIA patients.

Clinically, MIAT is positively correlated with lymphocytes in myocardial infarction patients and negatively correlated with neutrophils and platelets (Vausort, Wagner, and Devaux 2014). MIAT is positively correlated with IL‐6 and TNF‐α in patients with coronary heart disease and is an independent risk factor for coronary heart disease (Tan et al. 2019). This suggests that MIAT may be clinically associated with inflammation. Accordingly, a previous report has confirmed the close association between IA and inflammation (Jin et al. 2022). However, current studies have not found a relationship between MIAT expression and clinical characteristics of IA. This study revealed a significant correlation between MIAT expression and hypertension, location of aortic aneurysm, and aneurysm rupture. Logistic regression analysis demonstrated that MIAT expression can serve as an independent risk factor for poor prognosis in IA patients. IA patients with high expression of MIAT showed an increase in the rate of rupture, which significantly reduced the overall survival. Ruptured IA exhibits extracellular matrix degradation and elastic fiber degradation (Fan et al. 2023). Importantly, MIAT is a potential contributor to the extracellular matrix deposition mechanism (Chuang et al. 2020). Therefore, a potential explanation for the impact of MIAT on the prognosis of IA may be that MIAT overexpression may weaken the vascular wall by regulating the extracellular matrix, leading to IA rupture. Moreover, MIAT can function as a miRNA sponge through competing endogenous RNA (ceRNA) mechanism, and target downstream genes, thereby promoting the progression of cardiovascular diseases such as myocardial hypertrophy, atherosclerosis, and heart failure (Aonuma et al. 2022; Gao, Yue, and Huang 2021; Zhu et al. 2016). MIAT binds to RNA‐binding proteins to regulate downstream gene expression (Yang et al. 2022; Zhou 2022). The above findings provide potential research directions for the study of the molecular mechanism of MIAT in IA rupture. In the future, we will conduct more research on the molecular mechanism of MIAT in IA through cell and animal models, providing more theoretical basis.

There were still several limitations in our study. Firstly, our study is a single‐center study in a single ethnic group, with all participants being Chinese. Therefore, the results cannot be generalized to other ethnicities and may vary between different races. Secondly, the shape and morphological characteristics were not considered, and their relationship with MIAT expression was not clear. In future experiments, we will conduct a multi‐center prospective study to increase the size of samples and controls, thereby enhancing the reliability of the results. Additionally, we plan to investigate the regulatory mechanisms of MIAT by collecting basic experimental data at the cellular level and in animal models and to explore its potential regulatory role in the formation, rupture, and prognosis of IA.

As a prospective study, this research innovatively elucidates the high expression of MIAT in the serum of patients with IA. Our study has indicated that MIAT demonstrates high diagnostic value for IA, serves as an independent risk factor for IA rupture, and is associated with poor prognosis in IA patients. Thus, this study holds significant clinical relevance for the diagnosis of IA and prediction of IA rupture risk.

## Ethics Statement

This study received approval from the ethics committee of The Third Hospital of Inner Mongolia Autonomous Region, and all participants provided informed consent before enrollment.

## Author Contributions

**Fuquan Liu**: Conceptualization; investigation, writing—original draft. **Shuai Hao**: Formal analysis, investigation. **Jingliyu Wang**: Investigation. **Lei Chen**: Investigation. **Ning Jiang**: Resource. **Laixing Liu**: Investigation. **Xiangyi Wang**: Conceptualization, supervision, validation, writing—review and editing.

## Conflicts of Interest

The authors report there are no competing interests to declare.

### Peer Review

The peer review history for this article is available at https://publons.com/publon/10.1002/brb3.70500

## Data Availability

The datasets used and/or analyzed during the current study available from the corresponding author on reasonable request.
